# Point-Of-Care Testing Curriculum and Accreditation for Public Health—Enabling Preparedness, Response, and Higher Standards of Care at Points of Need

**DOI:** 10.3389/fpubh.2018.00385

**Published:** 2019-01-29

**Authors:** Gerald J. Kost, A. Zadran, L. Zadran, I. Ventura

**Affiliations:** Point-of-Care Testing Center for Teaching and Research, Pathology and Laboratory Medicine, School of Medicine, University of California, Davis, Davis, CA, United States

**Keywords:** point-of-care testing, curriculum, accreditation, public health, preparedness, standards of care, points of need, epidemic

## Abstract

**Objectives:** To develop awareness of benefits of point-of-care testing (POCT) education in schools of public health, to identify learning objectives for teaching POCT, to enable public health professionals and emergency responders to perform evidence-based diagnosis and triage effectively and efficiently at points of need, and to better improve future standards of care for public health practice, including in limited-resource settings and crisis situations.

**Methods:** We surveyed all U.S. schools of public health, colleges of public health, and public health schools accredited by the Council on Education in Public Health (CEPH). We included accredited public health programs, so that all states offering public health education were represented. We analyzed survey data, public health books, and board certification guidelines. We used PubMed to identify public health curriculum papers, and assessed 2019 CEPH accreditation requirements. We merged POCT knowledge bases to design a new curriculum for teaching public health students and practitioners the principles and practice of POCT.

**Results:** Public health curricula, certification requirements, and textbooks generally do not include POCT instruction. Only one book, *Global Point of Care: Strategies for Disasters, Emergencies, and Public Health Resilience*, and one online course on public health preparedness address POCT and public health intervention issues. The topic, POC HIV/HCV ED testing, appeared in one course and POC diagnostics in local clinics, in another. Papers on public health curriculum have not incorporated POCT. No curriculum addresses POCT in isolation units during quarantine, despite evidence that recent Ebola virus disease cases in the U.S. and elsewhere proved unequivocally the need for POCT. The modular learning objectives identified in this paper were customized for public health students. Public health graduates can use boot camps, online credentialing, and self-study to acquire POCT skills.

**Conclusions:** Enhancing accreditation requirements, academic training, board certification, and field experience will generate public health healthcare professionals who will rely upon evidence-based medical decision making at points of care, including during crises when time is of the essence. A POCT-enabled public health workforce can help prevent and stop outbreaks. Public health-based medical professionals urgently need the skills necessary to perform POCT and prepare America and other nations for threats portending significant adverse medical, economic, social, and cultural impact.

## Introduction

Point-of-care testing (POCT) is defined as diagnostic testing at or near the site of care (1). Catastrophic losses from the Ebola virus disease epidemic in West Africa and from other outbreaks, such as MERS-CoV in South Korea, reinforce the need for POCT. Educating public health practitioners to perform POCT directly at points of need will help control outbreaks of highly infectious threats. Avoiding costly pandemics will garner significant return on societal investments in teaching POCT in schools, colleges, and programs of public health. The U.S. and other nations that transiently enacted measures to enhance community resilience for Ebola virus disease during the 2014–16 epidemic and for other threats *remain ill prepared*. Centers for Disease Control and Prevention (CDC) funding remains inadequate to support preparation and response in limited-resource regions abroad.

The CDC estimates that an infectious disease outbreak in Southeast Asia could cost the U.S. economy up to $40 billion in export revenue and put almost 1.4 million U.S. jobs at risk (2). The Global Health Security Agenda was launched in 2014 to promote international collaboration among nearly 50 countries to prevent, detect, and quell infectious disease outbreaks. According to one CDC official, “The economic linkages between the U.S. and these global health priority countries illustrate the importance of ensuring that countries have the public health capacities needed to control outbreaks at their source before they become pandemics” (2). However, the CDC may be forced to narrow its countries of operation, real-time surveillance, and early detection, and this, despite the most recent outbreaks of Ebola virus disease in the Democratic Republic of the Congo.

Public health educators in the U.S. have not met the knowledge and skill levels needed for rapid response at points of care. Communities would be wise to develop countermeasures, such as immediately accessible diagnostic capabilities that enhance resilience in the event of contagion and quarantine, in part to ameliorate civil rights issues by supporting well thought-out and equitable care plans. POC-enabled public health concepts, knowledge, and skills must be codified by updating accreditation and certification requirements to include POCT. To help fill striking gaps discovered by our national survey, we designed multi-purpose curriculum topics and modular learning objectives that can be used to teach students and practitioners of public health the principles and practice of POCT.

## Methods and Materials

### Research Framework

We adopted a logical, systematic, integrated, and thorough research methodology leading to the POCT curriculum presented here. The research framework supports a harmonized curriculum practical for teaching in developed countries, such as the United States, Europe, and Japan, as well as in limited-resource countries, such as those in Africa and Asia. POCT has significant potential for preventing and limiting outbreaks of infectious diseases where they originate, if public health practitioners become educated in the principles and practice of POCT.

The Council on Education for Public Health (CEPH, https://ceph.org/) accredited (3) 64 schools of public health, colleges of public health, and public health schools (collectively abbreviated “SPHs”), and 117 public health programs (“PHPs”) in 2018 (4). CEPH accreditation lists provided the framework for systematic searches of curricula available online at SPH and PHPs in the U.S., including the District of Colombia. Four states, Delaware, South Dakota, Wyoming, and Vermont, do not have accredited SPH or PHPs. Five CEPH-accredited institutions outside the U.S. in Alberta Mexico, Montreal, Puerto Rico, and Taiwan were omitted.

### Survey Scope

The survey was administered to *all* schools of public health, colleges of public health, and public health schools (referred to as “SPH”) accredited by the CEPH in the U.S. After removing 5 foreign institutions from the 64 listed by the CEPH, the remaining 59 accredited SPH were contacted successfully (100% inclusive rate) online by accessing their websites, all of which posted current curriculum. These 59 are located in 33 states plus 1 in the District of Columbia. Institutions within individual states number: 5—NY; 4 each—CA and GA; 3 each—FL, MA, PA, and TX; 2 each—IN, KY, LA, MD, OH, OR, and TN; and 1 each—the remaining 19 states excluding DE, SD, VT, and WY, which have no SPHs or PHPs; plus 1 in Washington, DC.

To ensure geographic representation of all states, we included an additional 13 accredited public health programs (referred to as “PHPs”), in order that all states (100% inclusive rate) with public health educational institutions, that is, a total of (50 – 4 =) 46 states in the U.S., were represented. Multiple institutions in states with high populations balanced survey demography naturally. The total number of institutions surveyed was 72. The survey was completed in December, 2018.

### Knowledge Bases

Extracted online university bulletins, catalogs, course descriptions, course titles, syllabi, elective descriptions, learning objectives, lists of competencies, program guides, slide presentations, and other publicly available resources were archived and searched for acronyms (e.g., POCT) and key words, such as bedside testing, diagnostic testing, field testing, isolation, molecular diagnostics, pathogen detection, point-of-care testing, and reference laboratory, all compiled in a search dictionary used to assure consistency of researchers.

Tables of contents and indices of textbooks and reference books addressing public health issues were identified through Google, Amazon, and other online searches. Twelve highly ranking and relevant books were selected for tabulation. Inclusion criteria comprised Amazon rankings, public health content relevant to POCT, recent publication (2015 and later), and/or emphasis on global health, in view of the impact of POCT worldwide. Foreign language books were excluded.

The American Association for Clinical Chemistry (AACC, www.aacc.org) provided information about POC Coordinator credentialing courses found at https://www.aacc.org/store/certificate-programs/11700/point-of-care-specialist-certificate-program-2018 and https://www.aacc.org/store/certificate-programs/11700/improving-outcomes-through-point-of-care-testing-certificate-program-2018. We also investigated the learning objectives for a “boot camp” (5) intended for new POC operators. We tabulated separately a few representative online programs in public health, although information available at their websites was inadequate or non-existent for the purposes of this national survey.

The Centers for Disease Control and Prevention (CDC) offers a 1-h on-demand course in Clinical Laboratory Improvement Amendments'88 (CLIA) waived testing at https://www.cdc.gov/labtraining/training-courses/ready-set-test.html. Learning objectives are: a) to identify the CLIA requirements for performing waived testing, b) to follow the current manufacturer's instructions for the test, and c) to describe good testing practices to be used while performing waived tests (6, 7). The CDC is a Certified in Public Health (CPH) provider that offers 2.0 CPH recertification credits for completing this course. Although the course is dated, CDC insights were integrated into the public health curriculum we designed. Additionally, the curriculum includes instruction on the classification system (CLIA-waived, moderately complex, and complex) used by the FDA.

## Results

### Accreditation Requirements and Board Certification

We assessed CEPH accreditation criteria, using the “redline” version published on March 16, 2018 (3). POCT training is not listed among accreditation requirements for public health institutions. The National Board of Public Health Examiners (NBPHE, https://www.nbphe.org/) did not list POC concepts among the Certified Public Health (CPH) content outline (https://s3.amazonaws.com/nbphe-wp-production/app/uploads/2017/05/Content OutlineMay-21-2019.pdf) for 2019 (8).

### Professional Associations

The American Public Health Association (https://www.apha.org/) does not list POCT among topics and issues, not even in the “preparedness” category. We checked the Association of Public Health Laboratories and American Society for Microbiology (ASM) at https://www.cdc.gov/labtraining/external-partner-training.html. Neither currently posts POCT curricula, although the ASM and its Academy, in collaboration with the Infectious Disease Society of America, promotes webinars and general education in POC molecular diagnostics.

### POC Curriculum for Limited-Resource Nations

We drew on essential learning objectives in an original lecture (~500 slides) and workshop (laboratory) professional course for POC operators in limited-resource settings created by Kost et al. (9) in collaborations with a commercial diagnostics company, professional associations, and universities. This course has been taught worldwide (by its creators and other educators) over the past decade, typically in the format of two morning lectures followed by an afternoon hands-on workshop using capillary blood samples and POC devices that generate real-time results. Specifically, the curriculum has been taught and well-received in Canada, Europe, the Middle East, Africa, China, and SE Asia (Cambodia, Indonesia, Philippines, Thailand, and Vietnam), in the US for visiting scholars from Brazil, and in other countries.

### Monographs

Table 1 lists public health books. Only one, *Global Point of Care: Strategies for Disasters, Emergencies, and Public Health Resilience*, contributed by 105 authors and produced by the POCT•CTR (10), covers POCT topics relevant to public health and vice-versa, such as biohazards, community preparedness, disaster caches, geographic information systems, global resilience, national guidelines, molecular diagnostics (an extensive seven-chapter section), needs assessment, rapid diagnosis of critically ill patients with life-threatening diseases, POC culture, public health policy, and strategies for enhancing resilience.

**Table 1:** POCT content in one dozen representative public health books.

**Author(s), publisher, year, and ISBN**	**Book title**	**Pages, edition, cost, e-book, volume, series, appendices**	**POCT content**	**POCT chapters**
**R. Brownson, E. Baker, A. Deshpande, K. Gillespie** Oxford University Press 2017 ISBN-13: 9780190620936 ISBN-10: 0190620935	Evidence-Based Public Health	368 pages Third edition Paperback: $55.00 E-book: $52.25	None	None
**B. Clements, J. Casani** Butterworth-Heinemann 2016 ISBN-13: 9780128019801 ISBN-10: 0128019808	Disasters and Public Health: Planning and Response	538 pages Second edition Paperback: $83.95 E-book: $79.75	None	None
**R. Detels, M. Gulliford, Q. Karim, C. Tan** Oxford University Press 2015 ISBN-13: 9780199661756 ISBN-10: 0199661758	Oxford Textbook of Global Public Health	Three volumes 1,854 pages Sixth edition Hardcover $285.89 Paperback: $116.01!sathya! E-book: $138.57!sathya! Series: Oxford Textbook	None	None
**C. Holtz** Jones & Bartlett Learning 2016 ISBN-13: 9781284070668 ISBN-10: 1284070662	Global Health Care: Issues and Policies	644 pages Third edition Paperback: $78.95 E-book: $64.32	None	None
**G. Kost, C. Curtis** AACC Press-Elsevier 2015 ISBN-13: 978-1594251726 ISBN-10: 159425172X	Global Point of Care: Strategies for Emergencies, Disasters, and Public Health Resilience	701 pages First edition Appendices: 3 Special chapter: Understanding of POC culture improves resilience and standards of care in limited- resource countries	Extensive coverage of POCT and public health, including multiple chapter sections on community preparedness and resilience. Also, strategies, public health policy, and guidelines.	55
**J. Masci, E. Bass** CRC Press 2017 ISBN-13: 9781498717816 ISBN-10: 1498717810	Ebola: Clinical Patterns, Public Health Concerns	262 pages First edition Hardcover: $52.50 E-book: $77.73 Appendix	None	None
**M. Merson, R. Black, A. Mills** Jones & Bartlett Learning 2011 ISBN-13: 9780763785598 ISBN-10: 0763785598	Global Health: Diseases, Programs, Systems, and Policies	940 pages Third edition Hardcover: $74.30 E-book: $63.02	None (Includes a chapter on “Complex Emergencies”)	None
**R. Scheffler** World Scientific 2016 ISBN-13: 9789814612319 ISBN-10: 9814612316	World Scientific Handbook of Global Health Economics and Public Policy	1,628 pages First edition 3 volumes Hardcover: $1,001.44	None (Includes a chapter on “Technological Innovation in Health Care: A Global Perspective”)	None
**M. Schneider** Jones & Bartlett Learning 2016 ISBN-13: 9781284089233 ISBN-10: 1284089231	Introduction to Public Health	594 pages Fifth edition Paperback: $82.25 E-book: $54.09	None	None
**L. Shi, D. Singh** Jones & Bartlett Learning 2015 ISBN-13: 9781284100556 ISBN-10: 1284100553	Essentials of the U.S. Health Care System	402 pages Fourth edition Paperback: $72.55 E-book: $59.12 Appendix	None	None
**R. Skolnik** Jones & Bartlett Learning 2015 ISBN-13: 9781284050547 ISBN-10: 1284050548	Global Health 101	576 pages Third edition Paperback: $76.71!sathya! E-book: $50.43 (NEW E-BOOK) Series: Essential Public Health	“New diagnostics for TB: Xpert MTB/RIF” (p. 483)	None
**B. Turnock** Jones & Bartlett Learning 2015 ISBN-13: 9781284069419 ISBN-10: 1284069419	Public Health: What It Is and How It Works	454 pages Sixth edition Paperback: $67.08 E-book: $63.02	None	None

### Online Training

Topics, learning objectives, and lectures of the AACC online courses for POC operators, often used for credentialing of POC Coordinators who supervise bedside testing programs in U.S. hospitals, were found at the links in Methods. The basic course is recommended first before taking the clinical course. POCT•CTR authors contributed several topics to the second course. Certificates issued upon passing exams are recognized as evidence of competency in POCT. AACC educational programs are accessible for a fee, and hence, their use is limited. Additionally, they may be phased out in lieu of a new national board examination, passage of which will lead to board certification in POCT.

### Current Status

Table 2 shows that virtually no SPHs or PHPs curricula address POCT. A few of the course catalogs and bulletins describe limited laboratory training, mainly in bench microbiology. The purpose, design, instrumentation, operation, personnel, and training necessary to implement an isolation laboratory for highly infectious diseases were not mentioned. Community-based public health appeared in several of the curricula without describing the roles of self-testing, primary care POCT, pathogen detection, alternate care facilities, isolation laboratories, or field preparation. There was no discussion of Disaster Medical Assistance Teams (DMATs) or the contents of their National Disaster Medical System diagnostic instrument caches for use at points of need during crises.

**Table 2:** Point-of-care testing taught in U.S. Public Health Institutions.

**Geographic region City and State (number)**	**Schools/Colleges of public heatlh and public health programs (php)**	**POCT in core curriculum (yes/no)**	**POCT in electives (yes/no)**	**POCT content**
**NORTHWEST AND ALASKA (4)**				
Anchorage, AK	University of Alaska Anchorage (PHP)	No	No	None
Corvallis, OR	Oregon State University	No	No	None
Portland, OR	Oregon Health and Science University	No	No	None
Seattle, WA	University of Washington	No	No	None
**SOUTHWEST AND HAWAII (8)**				
Tucson, AZ	University of Arizona	No	No	None
Berkeley, CA	University of California, Berkeley	No	No	None
Loma Linda, CA	Loma Linda University	No	No	None
Los Angeles, CA	University of California, Los Angeles	No	No	None
San Diego, CA	San Diego State Univ.	No	No	IPH online^a^
Honolulu, HI	University of Hawaii (PHP)	No	No	None
Las Cruces, NM	New Mexico State University (PHP)	No	No	None
Reno, NV	University of Nevada (PHP)	No	No	None
**MOUNTAIN STATES** **(5)**				
Aurora, CO	Colorado SPH	No	No	None
Pocatello, ID	Idaho State University (PHP)	No	No	None
Missoula, MT	University of Montana (PHP)	No	No	None
Grand Forks, ND	University of North Dakota (PHP)	No	No	None
Salt Lake City, UT	University of Utah (PHP)	No	No	None
**NORTH CENTRAL (12)**				
Iowa City, IA	University of Iowa	No	No	None
Chicago, IL	University of Illinois	No	No	None
Bloomington, IN	Indiana University	No	No	None
Indianapolis, IN	Indiana University—Purdue University	No	No	None
Manhattan, KA	Kansas State Univ. (PHP)	No	No	None
Ann Arbor, MI	University of Michigan	No	No	None
Minneapolis, MN	University of Minnesota	No	No	None
Saint Louis, MO	Saint Louis University	No	No	None
Omaha, NE	University of Nebraska	No	No	None
Columbus, OH	Ohio State University	No	No	None
Kent, OH	Kent State University	No	No	None
Milwaukee, WI	University of Wisconsin			None
**SOUTH CENTRAL (5)**				
Hattiesburg, MS	University of Southern Mississippi (PHP)	No	No	None
Oklahoma City, OK	University of Oklahoma	No	No	None
College Station, TX	Texas A & M Health Science Center	No	No	None
Fort Worth, TX	University of North Texas	No	No	None
Houston, TX	University of Texas	No	No	None
**NORTHEAST (18)**				
New Haven, CT	Yale University	No	No	None
Amherst, MA	University of Massachusetts Amherst	No	No	None
Boston, MA	Boston University	No	SPH SB 808: “Merging Clinical and Population-Based Perspectives in Public Health Practice: Tension and Resolution”	POC Emergency Department education and testing for HIV/HCV
Boston, MA	Harvard University	No	No	None
Baltimore, MD	Johns Hopkins University	No	Intl. Hlth. 221.710.11	Limited^b^
College Park, MD	University of Maryland	No	No	None
Portland, ME	University of New England (PHP)	No	No	None
Durham, NH	University of New Hampshire (PHP)	No	No	None
Piscataway, NJ	Rutgers, The State University of New Jersey	No	No	None
Brooklyn, NY	SUNY Downstate	No	No	None
Buffalo, NY	University at Buffalo, SUNY	No	No	None
New York City, NY	Columbia University	No	No	None
New York City, NY	CUNY SPH	No	No	None
Rensselaer, NY	Univ. at Albany, SUNY	No	No	None
Philadelphia, PA	Drexel University	No	No	None
Philadelphia, PA	Temple University	No	No	None
Pittsburg, PA	University of Pittsburg	No	No	None
Providence, RI	Brown University	No	No	None
**SOUTHEAST (19)**				
Birmingham, AL	University of Alabama at Birmingham	No	No	None
Little Rock, AR	University of Arkansas	No	No	None
Gainesville, FL	University of Florida	No	No	None
Miami, FL	Florida International Univ.	No	No	None
Tampa, FL	University of South Florida	No	No	None
Athens, GA	University of Georgia	No	No	None
Atlanta, GA	Emory University	No	No	None
Atlanta, GA	Georgia State University	No	No	None
Savannah, GA	Georgia Southern Univ.	No	No	None
Lexington, KY	University of Kentucky	No	No	None
Louisville, KY	University of Louisville	No	No	None
New Orleans, LA	Louisiana State Univ.	No	No	None
New Orleans, LA	Tulane University	No	No	None
Chapel Hill, NC	University of North Carolina	No	No	None
Columbia, SC	University of South Carolina	No	No	None
Memphis, TN	University of Memphis	No	No	None
Johnson City, TN	East Tennessee State University	No	No	None
Charlottesville, VA	University of Virginia (PHP)	No	No	None
Morgantown, WV	West Virginia University	No	No	None
**DISTRICT OF COLUMBIA (1)**				
Washington, DC	George Washington University	No	No	None
**ONLINE (3)**				
Liberty University	Link 1 (below)	No	No	None
National Univ.	Link 2	Not accessible	None listed	Unknown
Public Health Online	Link 3	No	No	None

a *The Institute for Public Health (IPH) in the School of Public Health at San Diego State University offers three online POCT courses (produced by GK) as part of the Pacific EMPRINTS program under the title, “Point-of-Care Strategies for Disaster Preparedness,” at the IPH website (https://iph.sdsu.edu/courses/online.php). The fee for continuing education credit approved by the National Commission for Health Education Credentialing, Inc. (provider number 101840) is a nominal $5 for each course*.

b *The course description for “Designing Transformative Innovation for Global Health” (Department of International Health) lists “making…POC diagnostics more available in local clinics” as “potentially transformative for improving health and narrowing disparities.” However, the course learning objectives do not explicitly include POCT*.

*1. https://www.liberty.edu/online-at-liberty/master-of-public-health-health-promotion/?acode=D82994&subid=%252Buniversities%20%252Bfor%20%252Bpublic%20%252Bhealth&tfn=8558129919&msclkid=218df748e63e1dd3a9f1ad964e1d2593&gclid=CKG7_8j_0doCFRyDxQIdE0wPHQ&gclsrc=ds&dclid=CNycjcn_0doCFUqMaQodlOINHw*.

*2. https://info.nu.edu/health-care/public-health/?track=pm_bing_hc&utm_source=bing&utm_medium=cpc&utm_campaign=nu_psn_hc_bmm_nrlsa_ca&utm_content=[Content]&utm_term=%2Bschool+%2Bof+%2Bpublic+%2Bhealth&msclkid=7ac17d47d8c9154cd29a279975fa2702&gclid=CInQ5rKE0toCFUeExQId-VEL6g&gclsrc=ds*.

*3. https://www.publichealthonline.org/*.

San Diego State University SPH offers online POCT education produced by GK in association with Pacific EMPRINTS at the University of Hawaii (see Table 2). Courses comprise: Part I. Point-of-Care Strategies: Critical Care, Disaster Medicine, and Public Health Preparedness Worldwide, Part II. Point-of-Care Technologies Research Network (NIBIB, NIH), and Part III. Point-of-Care Strategies: Conclusions and Recommendations. These online courses are intended for public health personnel, physicians, nurses, pharmacists, dentists, veterinarians, laboratory managers, medical technologists, and emergency medical services personnel. The courses provide an introduction to POCT, discuss how POCT can be used for decision-making in acute care and rural settings, and summarize lessons have been learned about POCT from recent disasters. They conclude with recommendations for emergency preparedness using integrated POCT technologies. Continuing education credits are offered for these courses.

### Public Health Innovation

Based on the survey results, teaching experience, relevant chapters in *Global Point of Care: Strategies for Disaster, Emergencies, and Public Health Resilience* (10) and in *A Practical Guide to Global Point-of-Care Testing* (11) (the two most current POCT compendiums), and on the several knowledge bases described in Methods, we designed original topic-based learning objectives tailored specifically for public health students and practitioners (Table 3).

**Table 3:** POC curriculum and learning objectives for public health schools.

**Section and topics**	**Learning objectives**
**Part I**.	**Getting Started—The Mission**
Goals, objectives, and overview of uses in public health	• Define POCT as *testing at or near the site of care* and appreciate that the definition does not depend on the instrument format or size • Understand the fundamental goals and objectives of POCT for rapid and effective evidence-based decision making at points of need • Introduce situations where POCT has proven benefits for public health • Understand the importance of generating fast results, so that triage can be performed efficiently and immediately
**Part II**.	**Fundamental Principles and Practice of POC Testing**
**A. TECHNICAL**	
Needs assessment	• Develop competency in needs assessment for POC diagnostics in public health • Apply to healthcare settings limited-resource countries
Instrument formats, selection, and validation	• Recognize basic formats for disposable, handheld, portable, and transportable POC technologies that perform *in vitro* testing • Describe disposable POC tests, including smartphone modules, and their advantages, disadvantages, and marginal cost-effectiveness • Have the ability to select and validate the correct instruments
Non-invasive monitoring vs. *in vitro* diagnostic testing	• Consider the operating principles of non-invasive devices, namely pulse oximetry for monitoring of oxygen saturation, and continuous hemoglobin monitoring • Compare *in vivo, ex vivo*, and *in vitro* approaches and advantages
Specimen processing	• Contrast whole-blood vs. plasma analysis, also dry blots • Outline specimen processing and suitable sample types for testing in the field, primary care, and emergency room • Review special requirements associated with isolation laboratories
Quality assurance (QA), quality control (QC), and proficiency testing (PT)	• Identify “waived tests” under CLIA'88 and compare other POC tests • Know the definition and importance of quality assurance, including internal quality control and external quality assessment • Learn the five basic elements of the individualized quality control plan (IQCP), including environmental stress; how to customize QA, QC, and PT; and the importance of continuous quality improvement • Recognize confounding factors in diagnostic testing
Environmental stresses	• Overview the effects of environmental stresses on POC instruments and reagents, and how to avoid adverse consequences • Study methods for modulating environmental conditions for POC reagent storage and transportation
Multiplex molecular diagnostics	• Gain a basic appreciation of multiplex assays used for the detection of viruses, bacteria, and fungi, that is, pathogen detection • List advantages, disadvantages, costs, and limitations • Show examples of current POC disposable tests and instruments commercially available
**B. DESIGN AND BUILD**	
Design criteria	• Read WHO and other POC device performance specifications
Commercialization	• Understand custom POC test clusters, basic manufacturing requirements, commercialization processes, and timelines
Regulatory oversight	• Review routine FDA 510(K) clearance and pre-market approval (PMA) • Outline the FDA system of classification of diagnostic tests (i.e., CLIA-waived, moderately complex, and complex) and the criteria for home testing. • Assess the ramifications for implementation, personnel, and use
FDA and WHO emergency use declarations	• Study the process, legal requirements, and terms of FDA emergency use authorizations (EUAs) and WHO emergency use assessment and listings (EUALs) • Locate EUA and EUAL listings and documentation of tests on the web
Accreditation options	• Understand the definition of accreditation and why an organization would engage in it • Discuss the main considerations and steps leading to accreditation • Consider inspections options for POCT [e.g., College of American Pathologists, Joint Commission, and CMS (for waived testing)]
**C. PRACTICUM**
Device hands-on experience	• Demonstrate CLIA'88 waived and moderately complex POC tests • Learn how to perform common POC tests, how to operate mobile POC instruments, and security features (e.g., UN and PW) • Watch demonstration videos of transportable whole-blood analyzers and test clusters for critical care and support of patients in isolation
Results interpretation	• Use case studies to demonstrate how to interpret basic test results
Performance evaluation	• Attend a workshop illustrating POC performance evaluation, such as regression analysis, Bland-Altman plots, and locally-smoothed (LS) median absolute difference (“LS-MAD”) curves and maximum absolute difference (“LS-MaxAD”) curves
Trouble shooting	• Gain experience at trouble shooting POC tests and devices • See examples of error codes and how to respond to them
Establishing a POC program	• Understand the steps necessary to establish a successful POC testing program
**Part III**.	**Integration of POC and Public Health Expertise**
Roles of public health personnel and POC Coordinators	• Recognize the benefits of teamwork among public health practitioners, POC Coordinators, reference laboratories, and clinical laboratories • Develop personnel resources and a database of skill sets in advance of disasters, emergencies, complex crises, and epidemics • Understand that public health students and professionals could become POC Coordinators
Training, credentialing, and assuring competency	• List and analyze approaches to multidisciplinary credentialing • Specify requirements for maintaining competency and annual reviews • Learn how to document competency of Disaster Medical Assistance Teams (DMATs) and other first responders
**Part IV**.	**Health Maintenance and Non-communicable Diseases (examples)**
Pregnancy	• Explore sensitivity, timing, and interferences, and the technical differences in disposable urine tests vs. plasma assays
Prediabetes and diabetes	• Appreciate why plasma glucose standardization is necessary for consistent performance of blood glucose meters • Understand the role of POC HbA1c testing in the diagnosis and monitoring of prediabetes vs. diabetes • Develop patient plans for self-testing of capillary whole-blood glucose • Correlate prevalence, demographics, and public health implications of evidence-based POC diagnosis in poor and rich nations
Acute coronary syndromes and acute myocardial infarction	• Study Spatial Care Paths™ (SCPs) for rapid home rescue of patients with acute chest pain • Apply evidence-based medicine (EBM) and learn why current POC cardiac troponin (cTn) tests are limited to ruling in (not ruling out) acute myocardial infarction • Read about prehospital diagnosis using POC cTn on ambulances • Strive to use POC cTn in rural areas to eliminate social inequity by rapidly diagnosing acute myocardial infarction and starting intervention
**Part V**.	**Communicable Diseases (examples)**
HIV	• Appreciate the POC methods of screening for HIV, including pregnant women for prevention of transmission and algorithms for newborns • Study the advantages of simultaneous multiplex testing for concurrent diseases, such as TB
Influenza A and B	• Apply EBM principles to influenza testing and understand predictive values and their use from the viewpoint of the primary care physician • See examples of portable CLIA-waived instruments (Liat, Roche Diagnostics; Alere-i, Abbott) useful during flu season
Malaria	• Review new POC tests (e.g., fingerprick Ag *Plasmodium falciparum*) and uses in Africa and other endemic areas
Strep throat screening	• Review primary care practices related to screening • Understand necessary follow-up testing
Tuberculosis and resistance testing	• Cover instrumentation for TB diagnosis and resistance testing [e.g., the GeneXpert MTB/RIF test as a marker for multidrug resistant TB (MDR TB)], by drawing on the foregoing instruction in molecular diagnostics • Establish appropriate settings and conditions for testing • List and abate environmental stresses (e.g., temperature and dust)
**Part VI**.	**Geospatial Science and Geographic Information Systems (GISs)**
Small-world networks (SWNs)	• Define, illustrate, and analyze healthcare SWNs •Set the stage for community public health practice using POCT in optimized SWN healthcare delivery systems
GIS applications to health systems	• Explain how to set up and analyze a GIS • Establish SCPs within SWNs for rapid diagnosis and treatment •Assess the impact of GIS analysis of SWNs and SCPs •Integrate smartphone POCT-GIS for sentinel case tracking
**Part VII**.	**Preparedness for Outbreaks, Epidemics, and Isolation**
**A. LECTURES**
Dynamic evidence-based medicine	• Present sensitivity, specificity, and predictive values of POC tests •Point out that the false negative rate, *FN(t)*, is a function of time, and therefore, sensitivity and the ability to rule out disease are dynamic when testing patients with evolving infections
Ebola virus and other highly infectious diseases	• Document how the 2014–16 Ebola virus disease epidemic and cases entering the U.S. proved unequivocally the need for POCT •Overview how POCT could have curtailed the 2014–16 epidemic •Delineate POCT effectiveness in curtailing recurrent outbreaks in the DRC and the potential for empowering field response in war zones •Survey POC technologies available for Ebola virus disease and other high risk pathogens
**B. WORKSHOPS**
Personal protective equipment (PPE)	• Don PPE and practice performing POC tests, then doff the PPE and show that work was performed without personal exposure
Isolation laboratory and quarantine	• Be able to read floor plans, design an isolation laboratory, equip it with POCT, and route specimen workflow •Understand specifications for biosafety cabinets and limits to performing molecular diagnostics and POC tests within them •Identify special aspects of personnel training and protection
Spatial care paths™	• Demonstrate sentinel case discovery, 911 intent, and fastest rescue routes in healthcare SWNs •Place POCT to optimize efficiency and effectiveness
IQCP, its five key components, and plan design	• Practice designing individualized quality control plans (IQCP) • Remember the five components of the testing process: specimen, test system, reagent, environment, and testing personnel • Sketch out an IQCP for POCT in an isolation laboratory associated with a hospital and in an alternate care facility
**Part VIII**.	**Disasters, Emergencies, and Rapid Response**
Disaster caches	• List the test clusters in DMAT POCT caches, the three US sites of storage, personnel training, and regional deployment, including Alaska and Hawaii • Recognize necessary steps in opening and using the compact and larger laboratory caches, test clusters, and their different purposes • Review the basics of specimen collection and sample preparation, including for infectious diseases, under challenging field conditions • Recognize the analytical limitations of POCT under disaster conditions
Performance standards	• Establish QC criteria necessary to complete before using POC devices from caches in the field during emergencies and disasters • Develop backup procedures in case of QA failures • Know National Disaster Medical System routines for maintaining high levels of performance when using POCT from caches in the field
Telehealth	• Gain familiarity with field connectivity and telecommunications
Alternate care facilities	• Integrate DMAT resources with community alternate care facilities
Bioterrorism	• Be aware of major threats, methods of detection, containment
Emergency management and emergency operations centers	• Relate POC concepts to public health emergency management systems and structures • Integrate public health emergency operations centers as critical tools for responding to crises near points of need •Formulate appropriate POC test clusters to enhance local community resilience and coordinate them with regional POC resources, personnel, and caches
**Part IX**.	**Global Health and Limited-resource Settings**
Community resilience	• Present key published literature that shows how POCT adds to community resilience and value every day and during emergencies
Bedside and beyond	• Map the starting and ending points for public health ownership of POC diagnostics, monitoring, and therapeutics responsibilities
National essential diagnostics lists	• Strategize national essential diagnostics lists that concentrate on disease-specific resources • Study which diagnostics listed can be delivered at points of need • Identify how to use POCT to implement diagnostics lists cost-effectively from the standpoint of value for global health challenges
POC culture	• Appreciate the assimilation of point-of-need diagnosis into the daily lives of people as technologies permeate personalized medicine • Scan the spectrum of POCT from the home to primary care, to concierge medicine, and on to the ER, labor room, OR, and ICU
Case studies	• Illustrate value propositions for POCT in limited-resource countries in Asia, Africa, and other settings
**Part X**.	**POCT Standards, Policy, and Guidelines**
**A. LECTURES**
ISO framework	• Outline the purpose and contents of the International Organization for Standardization (ISO) 22870:2016, “Point-of-care testing: Requirements for quality and competence,” and associated standards (e.g. ISO 15189:2012, Medical laboratories)
CDC and WHO	• Review the guidelines and documents published by the CDC and WHO for POC needs, technologies, and public health response
General Accountability Office (GAO)	• Analyze recent General GAO reports, webcasts, and documents regarding POC technologies for epidemics, molecular diagnostics, and cost-effective healthcare systems in the U.S. and abroad
Global status	• Compare and contrast national POCT policy and guidelines that have been established in Malaysia and Thailand, and their advantages and shortcomings (e.g., lack of disaster POC)
**B. WORKSHOPS**
Procedures	• Understand the necessity for a set of written policies and procedures for POC testing • Be able to identify the core content of a policy or procedure
Policy and guidelines workshop	• Give learners the opportunity to draft an outline of the contents of national POCT policy and guidelines for a limited-resource country
**Part XI**.	**Project Management and POC Value Propositions**
Project management and the POC committee	• Understand the basic principles of project management • Consider how to analyze and effectively manage stakeholders • Understand the importance of the POC committee, anticipatory planning, and preparation for community projects
How to write a business case and develop value propositions	• Understand what information should be included in a business case • Be able to analyze the cost-effectiveness and value of POC testing • Identify key issues to address when implementing a new POC service
**Part XII**.	**Conclusions and Future Trends: The POC Vision**
Course summary	• Recap what we have learned and what we can do with our knowledge to improve public preparedness, response, and health outcomes
Learner presentations	• Have teams of learners share studies of POC applications with which they have personal experience or have gleaned from literature
Future vision	• Understand the role of POC technologies in future public health initiatives, disaster preparedness, and stopping spread of outbreaks of highly infectious diseases in America and other countries

This curriculum has been validated through decades of on-site teaching in both developed and limited-resource settings worldwide and has been used, either in whole or in part, for the development of national guidelines by ministries of public health, POC Coordinators who oversee POCT, online teaching, and other facets of professional POCT practice, as summarized in the two major compendiums above (10, 11) and in reference (1).

## Discussion

### POCT Public Health Curriculum

The curriculum we designed includes Part VIII, “Disaster Preparedness, Emergencies, and Rapid Response,” which heretofore, has never appeared in either public health schools nor in national POCT policy and guidelines (see Part X, Table 3), such as those implemented in Malaysia (12) and Thailand (13). Following Part I. Getting Started—the Mission, an introduction to the course, laboratorians could help teach Part II, particularly technical topics (Part II.A.) and the workshop practicum (Part II.C.). Faculty with field experience in POCT applications could teach “Health Maintenance and Non-communicable Diseases” (Part IV.) and “Communicable Diseases” (Part V.). Recent monographs (10, 11) provide the basic information for instructors and students alike.

Part VI. offers an overview of public health geospatial approaches important when positioning POCT in the community and coordinating it with spatial care paths™ in healthcare small-world networks. Parts VII and VIII are crucial to prepare future generations of interventional public health operatives, such as those working in the global health field covered in Part IX. The last two major didactic sections, Parts X and XI, could be covered in seminars. Faculty can establish their own POCT vision (Part XII) and engage in changing national accreditation standards to produce consistency of knowledge skills. The POCT curriculum for public health can be taught in a one quarter or semester course. Accompanying hands-on laboratories would enhance long-term educational recall.

### Needs Fulfillment

No investigators have previously identified the magnitude of the educational gaps revealed by our national survey results. The POC curriculum we designed fills gaps in public health education and attempts to initiate a dialogue leading to availability of POC-enabled public health practitioners worldwide. We found only one educational article intended for “health professional students” (at the University of Zimbabwe College of Health Sciences), (14) one for “training of nurses,” (15) and one for OB-GYN residents (16) that were relevant to POCT. Another group addressed internet-based interactive education as a potential venue for education, but not specifically public health (17). None of the papers targeted POCT curricula for public health. We believe all students of public health should have the opportunity to learn the principles and practice of POCT, especially those studying community health, epidemiology, or planning, policy, and surveillance, as well as physicians and nurses seeking public health degrees, intending to work in rural or limited-resource settings, or preparing to become first responders for disasters and outbreaks of highly infectious diseases.

### Editorial Leadership

A search using the website of the *American Journal of Public Health* (https://ajph.aphapublications.org/) returned no articles when using the term “point-of-care testing,” although “testing,” *per se*, appeared frequently, mainly with “HIV.” Similar results were found when searching the websites of the journal, *BioMed Central Public Health* (https://bmcpublichealth.biomedcentral.com/) and *Public Health*, which yielded only two articles, neither focused on POCT, *per se*. A PubMed search for “public health curriculum” produced 178 papers, none of which addressed POCT or related topics. Conversely, when PubMed recovered POCT papers, none addressed public health curriculum. We recommend that editors of public health journals initiate dialogue, produce focus issues, and encourage education in POCT.

### Accreditation Standards

POCT was not among the criteria that CEPH lists for accreditation (3), nor is it included in the NCEPH content for CPH Certification (8), as we recommend it should be (below). Therefore, absence of POCT instruction from the curricula for public health schools, colleges, and programs is not surprising. A direct approach for maturing the POC paradigm within the field of public health would be to revise accreditation requirements and include POCT explicitly in tables of contents and course descriptions for SPHs and PHPs. Some respite is offered to current public health practitioners through the free lectures and a training center described next.

### Free Access

The POCT•CTR offers over three dozen online instructional YouTube videos at https://www.youtube.com/user/POCTCTR/videos (accessed January, 2019). Additional academic speakers who address regional POC Coordinator groups can be found among the webinars offered by Whitehat Communications (https://whitehatcom.com/). San Diego State University SPH offers online POCT education with credit for nominal fees, as noted above. These resources, if tapped properly and collaboratively by public health institutions and societies, would be useful for education targeting public health issues. Extensive free instruction has been conducted over the past decade in lectures and workshops provided by one author (GK) and colleagues worldwide using the curriculum developed for limited-resource nations (9). Table 4 summarizes the topics. This experience was integrated into the recommended public health curriculum presented earlier in Table 3.

**Table 4:** Curriculum topics for POC specialists in limited-resource settings.

**Module (M) or Workshop (W)**	**Curriculum topic**
M.1.	An overview of POCT
M.2.	Regulatory oversight and accreditation options
M.3.	Establishing a POCT program
M.4.	Instrument selection and validation
M.5.	Policies and procedures
M.6.	Project management and planning
M.7.	Training and competency
M.8.	Analyzing quality
W.1.	How to write a business case
W.2.	POCT for cardiac care
W.3	POCT for critical care
W.4.	Diabetes POC on the wards and in outpatient settings
W.5.	POCT in the emergency department
W.6.	Training and project management
W.7.	POCT committee and stakeholders
W.8.	Preparedness for disasters, epidemics, and quarantine

### Training Center Opportunity

We suggest contacting the International Center for Point-of-Care Testing (http://www.flinders.edu.au/medicine/sites/point-of-care/) (accessed January, 2019) at Flinders University in South Australia directed by Professor Mark Shephard. Center leadership has described valuable information (18–21) on their unique training program, which as far as we know, is the only one of its kind in the world producing Master and Doctor of Philosophy graduates in POCT. Although this training program focuses mainly on POC solutions to health problems in rural Australia, chapters in the recent book (11) edited by its founder, Dr. Shephard, and chapters on POC preparedness for highly infectious diseases and disasters (22, 23) fed forward to augment our knowledge bases used to design the POCT curriculum for public health (Table 3).

### Limitations

The survey was web-based, so it is possible that POCT content was missed during the discovery phase, if taught, disseminated, or demonstrated in classes or electives that were not explicitly listed or investigated as search targets. Not all public health educational programs, *per se* (identified by “PHP” in Table 2), were included in the survey, but on the other hand, 100% of 59 U.S. schools of public health, colleges of public health, and public health schools accredited by the CEPH were accessed and assessed successfully, and 100% of the states in the U.S. with public health educational programs were represented. We focused the survey on the U.S. However, POCT has enabled national response and preparedness in developing countries and can be useful in public health institutions worldwide (10).

## Conclusions

### Establishing a Global Vision

Survey results identified one of the main root cause problems in the U.S., namely, accreditation requirements omit POCT. Our vision of future public health entails POCT-equipped practitioners moving to points of need with mobile diagnostic tools that will help stop outbreaks quickly, maintain isolation efficiently, and manage quarantine equitably. Mobile testing, rapid diagnosis, and rational triage will help thwart contagion arriving from abroad. Public health practice in general will benefit from well-trained personnel capable of administering POCT both in the community and primary care, and as needed, during disasters, complex emergencies, and national crises. POCT is growing exponentially on a global scale, so the public health field should embrace it to advance public health practice at points of need.

### Enabling Spatial Care Paths™

Table 5 summarizes several potential pathways of future progress that embrace rapidly evolving technologies and mold POC culture (24–27). Public health practitioners who serve communities and their medical constituencies, can do so directly with POCT, rather than indirectly and slowly following unnecessarily circuitous referrals to regional laboratories. Spatial Care Paths™ (28–30) can be designed to solve specific public health problems and meet demands during infectious disease outbreaks, such as the 2018 Ebola virus disease recurrence in Central Africa.

**Table 5:** Pathways forward—moving public health to points of need.

**Action base**	**Impact**
Accreditation requirements (root cause of deficiencies)	• Add instruction in POCT to CEPH curriculum and degree requirements for SPHs and PHPs • Systematically inspect class contents to fill gaps in root cause deficiencies
Board certification content	• Integrate POC concepts in the NBPHE CPH examination • Assess understanding of the principles and practice of POCT • Check recall of basics of interpreting POC test results • Establish competence in POC isolation laboratory design • Support essentials of the POC public health curriculum • Document POC preparedness for outbreaks and epidemics • Confirm understanding of disaster POC and personnel logistics
CDC	• Use online training and study documents on POCT
Community resilience	• Lead the design and organization of isolation laboratories, POC device selection, personnel coordination, and alternate care facilities • Train staff to use personal protective equipment (PPE) and to operate POC devices while wearing PPE • Archive public health skill sets and plans for immediate access during disasters, crises, epidemics, and outbreaks
Experience abroad	• Seek internships and training in foreign countries in need of rapid response medical decision making, especially limited-resource nations
GAO	• Study reports on molecular diagnostics, infectious diseases, and POC diagnostics, and on their relationships to national need and impact
IFCC POCT Task Force	• Make use of educational documents and focused symposia found at http://www.ifcc.org/ifcc-education-division/emd-committees/taskforcepoct/poctresources/
Literature	• Use PubMed to locate, then read literature on POCT
NAS	• Study documents on bioterrorism and threat detection
NIH	• “Grand Challenge” for POC devices that detect antimicrobial resistance (NIAID, BARDA), POC technologies centers (NIBIB), and other point-of-need awards
Online training and boot camps	• Obtain individual AACC certifications in basic and clinical POCT • Obtain certificates or board certification by online examination • Enroll in “boot camps” for the POC Coordinator's perspective
Outreach	• Participate in community health fairs that promote self-testing for diabetes, HIV, pregnancy, and other communicable and non-communicable diseases
Promotion of public health schools	• Advertise training in POCT, PPE, isolation laboratories, and community-based POC diagnostics as a calling cards to attract learners to public health schools and programs
Public health books	• Include content on POCT and specialized applications, such as POCT for complex emergencies, disasters, and epidemics
Public health journals	• Fill the gaps in editorial leadership by recognizing the importance and impact of POCT for community response and resilience • Create a dialogue in public health journals globally with editorials, examples of implementations, and training cases • Organize feature issues of journals that focus on POCT
Special interest groups	• Form regional clubs, host POC speakers, and engage online webinars for sessions broadcast to the public health student body and local forums of practitioners
Vision	• Move public health to points of need using POCT
Whitehat Communications	• Participate in POC Coordinator webinars, live and archived (see https://www.whitehatcom.com/POC_Group_Webinars_2018.htm)
YouTube	• Learn from online lectures, such as the POCT•CTR series of presentations at https://www.youtube.com/user/POCTCTR

*AACC, American Association for Clinical Chemistry; AJPH, American Journal of Public Health; BARDA, Biomedical Advanced Research and Development Authority; CDC, Centers for Disease Control and Prevention; CEPH, Council on Education for Public Health; CPH, Certified Public Health; GAO, Government Accountability Office; NAS, National Academy of Science; NBPHE, National Board of Public Health Examiners; NIAID, National Institute of Allergies and Infectious Diseases; NIBIB, National Institute of Biomedical Imaging and Bioengineering; NIH, National Institutes of Health; IFCC, International Federation for Clinical Chemistry; and POCT•CTR™, Point-of-care Testing Center for Teaching and Research*.

### Acting Urgently

The American Academy of Microbiologists recommends, “Ensure that public health surveillance of infectious diseases is maintained with POC testing” (31). Some, such as pathologists, have been labeled “invisible,” that is, not providing adequate leadership, when it comes to, “Modern, affordable pathology and laboratory medicine systems…essential to achieve the 2030 Sustainable Development Goals for health in low-income and middle-income countries” (32–34). Academic leaders in China see such strong need for POCT, that they and the author (GK) have proposed a new field, “*Point of Careology*,” with its own dedicated professional staff. Calls for action originate from urgent needs. If SPH and PHPs start now, then given the lag in attaining and matriculating MPH and PhD degrees, the public health workforce will emerge better trained in POCT, hopefully, not too late.

## Public Health Implications

### Benefitting From Integrated Expertise

Enhanced public health education will promote cross-talk among medical specialties and help disperse knowledge necessary for rapid response and immediate decision making, especially to prepare hard hit regions like West Africa for new outbreaks of highly infectious threats. Management of the Ebola virus disease cases that appeared in the U.S. proved unequivocally the need for POCT and trained operators familiar with professional protective equipment, isolation laboratory designs, containment protocols, and suitable POC technologies (22, 28–30). Besides remedying educational deficits, public health leadership should engage in research regarding POCT optimization for isolation, mobile facilities, and the community.

Additionally, POCT for infectious diseases represents a means for pharmacists and public health professionals to collaboratively combat antibiotic resistance and improve community health (35, 36). While in need of POC education and curricula (37), which could be adapted from Tables 3 and 4, pharmacists appear amenable to using POC tests (38), including in low- and middle-income settings (39). Future research should assess the potential for nurses, physician assistants, and other professionals to become part of POC-enabled teams.

### Enhancing Accreditation and Certification Requirements

Public health leaders and the CEPH could create a consensus strategy for incremental assimilation beginning with a POCT course and accompanying hands-on workshop. National board exams are intended to protect the public from non-competency. The NBPHE should update CPH certification content consistent with future progress in education. Public health professionals could take the board certification exam possibly to be offered by the American Association for Clinical Chemistry. Becoming board certified will help produce consistent POCT performance and obviate surprising neglect of POCT evident, for example, even in a recent re-design (40) of public health school curriculum.

### Improving Standards of Care

POCT-trained public health practitioners will better prepare society for crises, while also delivering diagnoses and primary treatment directly to people in need. Distributed POC knowledge will help answer calls for action in the U.S. and abroad. The technology of diagnostics is changing rapidly, supported by progress in information technology, network analysis (41), geospatial sciences (42), and molecular diagnostics (43).

Like the rapid evolution and adoption of smartphones, POCT has become ubiquitous worldwide during the past decade and will continue to expand its horizons. In fact, several modular diagnostic tests (e.g., glucose, HbA1c, and infectious diseases) are in the commercial pipeline or already appearing for implementation on smartphone platforms, with which most, if not all, public health practitioners are familiar. Smartphones provide excellent connectivity and networking options of value to communicating test results and geospatially tracking sentinel patient cases.

Two recent books, *Essentials of Public Health Preparedness and Emergency Management* (44) *and Public Health Emergency Preparedness: A Practical Approach for the Real World* (45), position public health practitioners as first responders and promote them in that key role. However, neither book includes sections on POCT, training for it, or didactic curriculum. Neither addresses related topics, such as selecting POC devices for isolators or isolation units in support of critically ill patients with highly infectious diseases, although on p. 183 of reference 45 a brief case study by C. Standley about the 2014 Ebola outbreak in Guinea lauds POCT as a technological advance that “contributed significantly” to slow contagion. Clearly, a sturdier educational bridge should constructed to connect the point of care and public health fields.

Therefore, to improve standards of care in public health, public health educational institutions in the U.S. would be well-advised to integrate and teach POCT in formal accredited curricula and share teaching experiences with other countries, especially those at risk (46) from highly infectious diseases.

## Author Contributions

GK designed the research, completed Tables 1 and 2, compiled Tables 3–5, analyzed results, and wrote the paper. Three student researchers (AZ, LZ, IV) a) contributed and updated Table 1 and validated Table 2 by double checking all SPH/PHP websites, b) reviewed and helped correct the manuscript prior to publication, and c) approved it in its final form.

### Conflict of Interest Statement

The authors declare that the research was conducted in the absence of any commercial or financial relationships that could be construed as a potential conflict of interest.
